# The Sky Has Its Limits in COVID-19 Testing

**DOI:** 10.5041/RMMJ.10412

**Published:** 2020-07-31

**Authors:** Shai Linn, Shay Tzafrir, Shay Gueron

**Affiliations:** 1 School of Public Health, The University of Haifa, Haifa, Israel; 2 School of Management, The University of Haifa, Haifa, Israel; 3 Department of Mathematics, The University of Haifa, Haifa, Israel

**Keywords:** Bayes’ theorem, COVID-19, diagnostic tests, epidemiology, flights, methods, screening

## Abstract

At the time of writing, in July 2020, the COVID-19 pandemic has already inflicted dramatic international restrictions, including airports closing and limiting international travel. It has been suggested that re-opening of airports should involve and even rely on testing travelers for COVID-19. This paper discusses the methodology of estimating the detection and diagnostic accuracy of COVID-19 tests. It explains the clear distinction between the technical characteristics of the tests, the detection measures, and the diagnostic measures that have clinical and public health implications. It demonstrates the importance of the prevalence of COVID-19 in terms of determining the ability of a test to yield a diagnosis. We explain the methodology of evaluating diagnostic tests, using the predictive summary index (PSI), and the minimum number of tests that need to be performed in order to correctly diagnose one person, which is estimated by 1/PSI. In a population with low prevalence, even a high-sensitivity test may lead to a high percentage of false positive diagnoses, resulting in the need for multiple high-cost tests to achieve a correct diagnosis. Thus, basing a policy for opening airports on diagnostic testing, even with the best test for COVID-19, has some limits.

## INTRODUCTON: WHY DO WE NEED TO STUDY TEST ACCURACY?

At the time of writing, in July 2020, more than 13 million persons worldwide have been infected by the severe acute respiratory syndrome coronavirus 2 (SARS-CoV-2), leading to a pandemic of coronavirus disease 2019 (COVID-19). More than half a million persons have died of the disease.^1^ The accurate detection of the viral infection by medical and laboratory tests is critical for finding a cure and for planning public response measures. However, the results that the tests provide depend on the prevalence of the disease, as is explained below.

All medical tests are inaccurate, i.e. they may yield both false positive and false negative results. Some of these errors may have grave consequences for individuals and society. A false negative diagnosis may lead a person to be falsely assured and therefore avoid necessary quarantine. On the other hand, a false positive diagnosis may lead to unnecessary quarantine or unnecessary treatment.

We discuss the properties of the associated calculations, the accuracy measures of medical tests, and the estimate of the minimum number of tests that need to be performed in order to correctly diagnose one patient.

## TWO TYPES OF TESTS FOR COVID-19

There are two main types of tests used for detecting and diagnosing COVID-19 infection for clinical or surveillance purposes: molecular and serological.^2^–^4^

*Molecular tests* detect the pathogen of acute disease. These tests may also detect fragments of the pathogen before it is fully cleared from the body, even if the pathogen is no longer able to replicate or cause disease. Currently, such tests rely on a technique called reverse transcriptase–polymerase chain reaction (RT-PCR) to detect the presence of the virus. These tests can provide data on the incidence of the disease, i.e. the fraction of a population that is newly infected. This paper is focused on the evaluation of these tests.

*Serological tests*—sometimes referred to as “antibody tests”—can provide information about the prior infection of an individual, indicating the body’s immune response to the virus, and can detect the infection after convalescence. These tests can provide data on the prevalence of the disease, i.e. the fraction of the population that has been infected by the disease in the past. The body makes IgM and then IgG antibodies within about 10 days; IgM indicates a very recent infection, and IgG indicates infection at any time in the past. Serological tests can help public health officials determine the prevalence of previous infection, including among asymptomatic individuals, and among those with mild symptoms who did not seek medical care. Prevalence determination may help in deciding to relax social distancing and quarantine measures. Further, the serological test may be used to explore an individual’s previous infection by the virus. This paper explains the importance of utilizing the information from serological tests, i.e. for determining the prevalence of the disease.

## TWO TYPES OF TEST ACCURACY MEASURES

Each of the above tests may be used for two different goals: *detection* (Table 1) or *diagnosis* (Table 2) of the disease.^5^–^21^

**Table 1:**
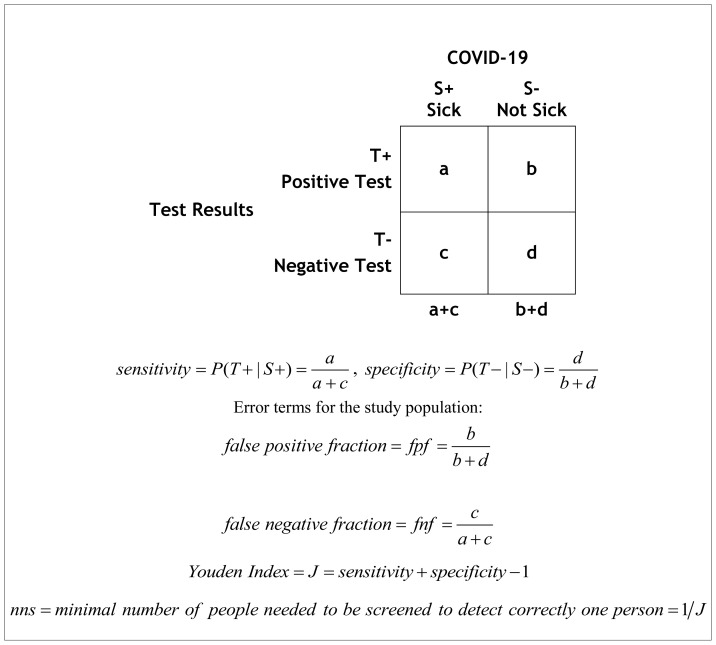
Data Presentation in a Selected Population, Assessing the Detection Capability of a Test.

**Table 2:**
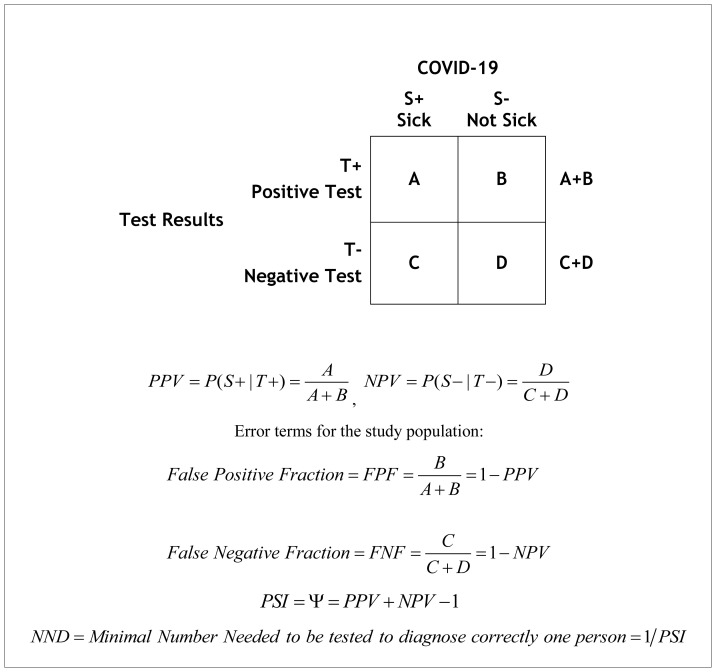
Data Presentation in a Clinical Study Setting in a Target Patient Population Assessing the Diagnostic Capability of a Test.

### Detectability Measures: Technical Characteristics of No Clinical Importance

We use italic lower-case letters in the description of screening in the general population in a 2×2 table, Table 1. The *sensitivity* and *specificity* are calculated in samples of persons with (a+c) and without (b+d) the disease in a selected population. In this table, it is not appropriate to include the totals of the “horizontal” axis of test (T) results.

A researcher could determine the detectability of COVID-19 in a study where the prevalence of the disease is *artificial*. For example, a study may calculate the *sensitivity* and *specificity* of a test in 100 persons with a disease (e.g. clinical COVID-19), and 100 persons without the disease. We set the prevalence of COVID-19 in this particular study, artificially, to be 50%.

The *sensitivity* and *specificity* are used to describe the *technical* characteristics of a test. These measures are not useful in the clinical setting, because the prevalence of the disease is different in a true patient population. The sensitivity or specificity could tell us the *percentage* of the persons with (or without) the disease that would be detected, but we will not know *how many* patients with COVID-19 (or without it) will be diagnosed correctly. For example, we could know the percentage of persons with the disease that would be quarantined based on detection by a test, but we could not know how many persons would be quarantined. The information on the percentage of detected persons would be meaningful only if we know the prevalence of the disease.

The fraction (percent) of persons with the disease who would not be identified (i.e. the false negative fraction) is *fnf* = 1 – *sensitivity*. The fraction (percent) of persons without the disease who would not be identified (i.e. the false positive fraction) is *fpf* = 1 – *specificity*.

The Youden index (*J*) is a summary measure of the goodness of a test. It describes the percent of correct detection (without false negative nor false positive detection). This index is defined as:

(Eq. 1) $$\begin{array}{l} J=1-(fpf+fnf)\\ =1-[(1-sensitivity)+(1-specificity)]\\ =sensitivity+specificity-1 \end{array}$$

When *J*=1, the test is always correct: there are no errors, so *fpf* + *fnf* = 0, and the test detects correctly the sickness status.

When *J*=0, assuming that *sensitivity* and *specificity* are of equal importance in determining the expected gain, the test provides no information. In other words, the test is useless if the proportion of errors equals 100%, and *fpf* + *fnf* = 1, leading to *J*=0.

When −1 < *J* < 0, the test is misleading: its results are negatively associated with a true diagnosis. When *J* = −1, the test is always misleading.

*J* can also be interpreted as the difference between the true and false positive fractions.

Since *J* = *sensitivity* – *fpf*, *J* reflects the *excess* of the proportion of a *positive* result among patients with the disease versus patients without the disease. Similarly, *J* also reflects the excess in the proportion of a *negative* result among patients without the disease versus patients with the disease. This can also be written as *J* = *specificity* – *fpf*.

#### J as a difference measure of detectability analogies to a cohort study

Table 1 is analogous to a clinical trial or a cohort study that compares the risk of a disease among those exposed to a risk factor, *R_exposed_*, and the risk among those who are not exposed, *R_non-exposed_*. The “causative” variable (i.e. the “exposure”) is the fact that a person does or does not have the disease, and the diagnostic test results (positive or negative) are the “outcome” of the disease. The difference in risk between the exposed and non-exposed persons is measured by the risk difference (*RD*):

(Eq. 2) $$\begin{array}{l} RD=Risk\;difference\\ =R_{exposed}-R_{non-exposed} \end{array}$$

*J* is analogous to *RD*.

(Eq. 3) J=sensitivity-fpf

Therefore, a derived analogy of the well-known measure of the “number needed to treat”, (*NNT*) = 1/*RD*, is 1/*J*. The value 1/*J* may be interpreted as the number of persons that need to be examined in order to correctly detect by screening (*nns*) one person with the disease (Table 1) of persons with and without the known disease. The *nns* could help in estimating the minimum number of tests that has to be applied to persons with *known* diagnosis of COVID-19 (with or without the disease) in order to detect one person correctly (positive or negative, respectively). It can be useful in assessing a *percent* of a successful monitoring program (how many of the persons with, or without, the disease will be detected). However, it cannot assess *how many* persons with or without the disease will be detected, and thus it has no clinical or public health importance, because it cannot be applied to a real population in which we do not know the COVID-19 diagnoses.

Currently, PCR tests have a sensitivity or specificity of approximately 70%–95%, depending on the conditions of the tests.^5^ For example, the sensitivity of the PCR test using a nasopharyngeal swab is higher than that using a nasal swab, while the specificity of the test is lower using a nasopharyngeal swab. For simplicity, we assume here a 90% sensitivity and 90% specificity for both PCR and serological tests.

#### Example

Suppose that a population of 1000 travelers is screened for COVID-19, using the PCR test. The test will detect 90% of the persons with COVID-19. These persons will be treated or quarantined. However, the test will not detect 10% of the persons with the disease, i.e. the test will have an *fnf* of 10%. This would allow 10% of the persons with COVID-19 to continue interacting with their family and the community, with the implied risk of transmitting the disease. Similarly, the test would correctly detect 90% of the persons without COVID-19. These persons would not be quarantined. However the test would incorrectly detect infection in 10% of the uninfected persons, i.e. the test will have an *fpf* of 10%. This would allow 10% of the persons without COVID-19 to be unjustifiably quarantined. For these data, *J* would be 0.80. This indicates 80% better information compared to the case where no test is used, or if a useless test with *J*=0 is used. Note that we know only the test’s ability to detect the percentage, and not the number of persons that would be diagnosed with or without the disease. We know that the test will detect 90% of the persons with (or without) the disease. However, since the prevalence of the disease in the traveler population is not known, we will not know the number of persons with the disease (or without the disease, respectively). The test would not show how many travelers would need to be quarantined, nor how many travelers would not be quarantined because of false negative test results. Based solely on detection measures, it is not possible to plan quarantine facilities or to assess the number of persons with COVID-19 who are wrongly released into the community and continue to infect others. Thus, the detection measures cannot be useful for practical planning of public health measures. By contrast, diagnostic measures, which are explained below, can be used for these purposes.

### Diagnostic Measures of Clinical and Public Health Importance

The application of a diagnostic test to a patient (target) population utilizes a 2×2 table (Table 2). To evaluate the effectiveness of the application of a diagnostic test in the patient population, the investigator first observes the outcome, i.e. the test results, and obtains information about the study factor, i.e. the disease status.

We use *upper-case letters* to describe screening in the patient population in a 2×2 table, Table 2. It is the data in this table that are of interest to the patient (and the physician) and public health officials, answering the following questions: (1) When the test is positive, what is the probability that the patient has the disease? (answerable by the positive predictive value [*PPV*]); (2) When the test is negative, what is the probability that the patient does not have the disease? (answerable by the negative predictive value [*NPV*]).

In this clinical setting, the diagnoses are as yet unknown, and the test is used to diagnose COVID-19 in individuals: the *PPV* and *NPV* are an estimate of the test’s ability to diagnose patients accurately in a population (based on the real disease prevalence), i.e. of the fractions of patients who are *diagnosed correctly* as positive or negative, respectively. The *PPV* is the fraction (percent) of the positive tests in a given population that will correctly diagnose a COVID-19 patient. Similarly, *NPV* is the fraction (percent) of negative tests that will correctly diagnose a person who is not infected. The fraction (percent) of persons with a positive test who would not have the disease is the diagnostic false positive fraction (*FPF*), that is calculable as *FPF* = 1 − *PPV*. The fraction (percent) with a negative test result who have the disease and is diagnosed incorrectly as not having the disease is the diagnostic false negative fraction (*FNF*) calculable as *FNF* = 1 − *NPV*.

Both *PPV* and *NPV* depend on the proportion of the population that has the disease according to clinical or serological criteria, i.e. the *prevalence* of the disease. Thus, the *PPV* and *NPV* provide insight into the expected accuracy of the positive and the negative test results in a given population, by factoring in the ability of the test to detect the disease and the prevalence of the disease in the population.

Suppose that the same test were used in two different populations: population A with a higher disease prevalence and population B with a lower disease prevalence. Then, the *PPV* would be higher in population A than in population B, because the number of false positives would be a lower percentage of the total number of positive tests in population A. Similarly, the *NPV* would be higher in population A than in population B.

#### Predictive summary index as a summary measure of diagnostic ability of a test in individuals

A summary index, the predictive summary index (*PSI*, Ψ), is a measure of the additional information given by the test results, beyond the prior knowledge (the prevalence of the disease).^21^ Note that the information from a positive test result beyond what is already known about the disease prevalence is *PPV* – *Prevalence*. Similarly, the information from a negative test result beyond what is already known about the probability of no disease (the prevalence of no disease) is *NPV* – (1 – *Prevalence*).

Thus, the overall information, i.e. the gain in certainty obtained after a test is performed, beyond what is already known, can be calculated as a summary measure:

(Eq. 4) $$\begin{array}{l} Total\;gain\;in\;certainty=\\ =\left[PPV-Prevalence\right]+\\ +\left[NPV-(1-Prevalence)\right]=\\ =PPV+NPV-1=PSI \end{array}$$

Alternatively, Ψ is a summary of the information that is not derived from errors, *FNF* and *FPF*:

(Eq. 5) $$\begin{array}{l} PSI=1-(FPF+FNF)=\\ =1-[(1-PPV)+(1-NPV)]=\\ =PPV+NPV-1 \end{array}$$

If Ψ=1, the test is always correct: there are no errors, so that *FPF* + *FNF* = 0; i.e. the test detects correctly the sickness status. When *PSI* = 0, *PPV* + *NPV* = 1, and the test provides no overall information. In other words, the test is useless if the proportion of errors equals 100%; i.e. when *FPF* + *FNF* = 1, *PSI* = 0. For example, if the test results are random and the probability of both *PPV* and *NPV* is 50%, then the test is useless and *PSI*=0. When −1 < *PSI* < 0 the test is misleading; i.e. the tests results are negatively associated with the true diagnosis. When Ψ = −1, the test is always misleading.

The *PSI* can also be interpreted as the gained probability of correct diagnosis information, i.e. the difference between the joint probabilities of correct diagnosis (positive or negative diagnosis) PPV*NPV and the joint probabilities of incorrect diagnosis FPF*FNF:

(Eq. 6) $$\begin{array}{l} PPV*NPV-FNF*FPF=\\ =PPV*NPV-\left[\left(1-PPV)*(1-NPV\right)\right]=\\ =PPV*NPV-1+NPV+\\ +PPV-NPV*PPV=\Psi \end{array}$$

#### PSI as a difference measure of a diagnostic test

The *PSI* can be interpreted as the difference between the correct prediction of a disease by the test and a false negative result of the test in the target population.

(Eq. 7) PSI=PPV-FNF

Thus, *PSI* reflects the excess in the proportion of infected people in those with a positive result versus the proportion of infected people when the test is (falsely) negative.

Similarly, one can also interpret *PSI* as

(Eq. 8) PSI=NPV-FPF

Here, *PSI* reflects the excess in the proportion of uninfected persons when the test yields a negative result versus the proportion of uninfected people when the test is (falsely) positive.

The *NND* = 1/*PSI* is analogous to *nns*, to estimate the number of patients who need to be examined in the patient population, in order to correctly diagnose one person (see Table 2). For example, this can be the number of people who would have to undergo a PCR test to correctly diagnose one person. This measure may be abbreviated as the “number needed to diagnose” (*NND*). This information has public health importance. It also enables planning of test services to a specific population, based on the prevalence of the disease in this population as well as on the technical characteristics of the test.

## CALCULATING THE DIAGNOSTIC ACCURACY MEASURES USING THE PREVALENCE AND THE DETECTION ACCURACY MEASURES

The data in Table 1 are usually provided by the manufacturer of a medical test, or obtained in a study with a sample of persons with or without the disease. Such a study does not reflect the prevalence of the disease in the general population because the ratio of the number of persons with the disease to the number of persons without it is artificially determined by the researcher. However, the data in a study that samples persons with positive or negative test results (Table 2) are often unavailable because it is impossible or unethical to follow up persons with negative diagnostic test results. Thus it is necessary to calculate *PPV* and *NPV* from the data in Table 1, using the true prevalence of the disease.

Such calculations can be performed using Bayes’ Theorem (see below for Eq. 9).^4^–^21^

(Eq. 9) $$\begin{array}{l} PPV=\frac{sensitivity*Prevalence}{sensitivity*Prevalence+(1-specificity)\;(1-Prevalence)}\\ =\frac{sensitivity*Prevalence}{sensitivity*Prevalence+fpf*(1-Prevalence)} \end{array}$$

Using *sensitivity*=90%, *specificity*=90%, and *Prevalence*=1%, we get *PPV*=8.3%.

A similar equation exists for calculating *NPV*.

Alternatively, a table that reflects the real prevalence of the disease can be constructed, using the sensitivity and specificity, enabling *PPV* and *NPV* calculation (Table 3 in the following example).

**Table 3:**
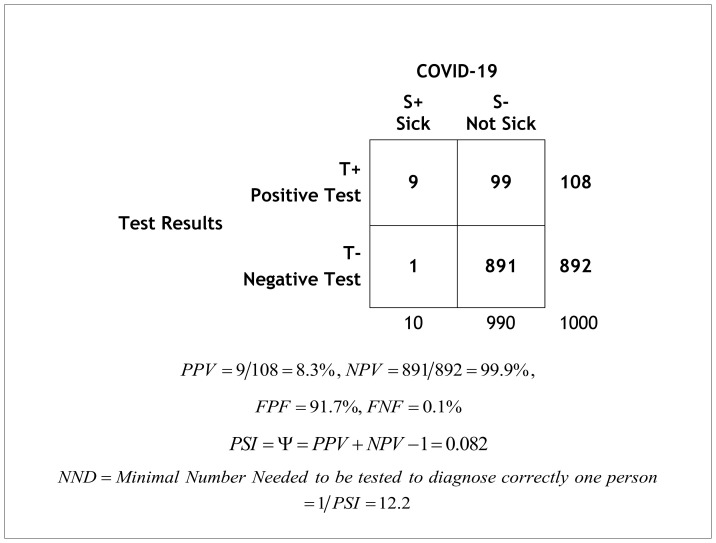
PPV, NPV, PSI, and NND calculated based on the Prevalence 1%, Sensitivity 90%, and Specificity 90%.

### Example

The following is an example for calculating *PPV*, *NPV*, *PSI*, and *NND* using data on the prevalence of the disease and given data on the sensitivity and specificity of the test (Table 3).

Suppose that a population of 1000 travelers is screened for COVID-19 using the PCR test, and that the prevalence of COVID-19 is 1% (which is an estimated prevalence of asymptomatic persons in Israel who had been exposed to a person with COVID-19). Of the 1000 travelers, only 10 are COVID-19 patients, and 990 are free of the disease. If the sensitivity is 90%, then 9 persons will be correctly diagnosed as infected with COVID-19 (and 1 person will not be diagnosed, false negative). If the specificity is 90%, only 891 persons will be diagnosed correctly as free of COVID-19, but 99 travelers will be falsely diagnosed as having the disease and will be quarantined unnecessarily. The *PPV* is only 9/108= 8.3%, and the *FPF*=91.7%. Thus, the fraction of false positive diagnoses is very high, and many persons will be quarantined or denied a flight unnecessarily. On the other hand, the *NPV* is 99.9%, and there are only a few false negative diagnoses. The test will be very useful to diagnose person who are not sick with COVID-19, and may be helpful to ensure that passengers are without COVID-19. The *PSI* is 8.2%, which is a very low proportion of additional information obtained by the test. However, this is very useful information. Indeed, more than 12 tests will be needed to diagnose one person correctly, because of the high percentage of false positive tests. There were more than a billion United States passengers flying every year before the corona pandemic. Thus, the tests may be very expensive, and will impose significant personal hardship to persons with false positive diagnoses. However, it will be a useful test in order to make sure that no person with COVID-19 will board a flight.

### *NND* with a Changing Prevalence, Where Sensitivity and Specificity Equal 90%

Table 4 summarizes *NND* with a changing prevalence, where sensitivity and specificity are 90%. The *PPV* is increasing and the *NPV* is decreasing with increasing prevalence. When the COVID-19 prevalence is 1%, 5%, or 10% (most estimates in different countries and societies are in this range), more than 12, 3, and 2 tests, respectively, are needed to diagnose one person correctly.

**Table 4:** Example for *PPV*, *NPV*, *PSI*, and *NND* as a Function of the Prevalence with 90% Sensitivity and Specificity.*

Prevalence (%)	Estimated Prevalence	*PPV* (%)	*FPF* (%)	*NPV* (%)	*FNF* (%)	*PSI* (%)	*NND*=1/*PSI*
1	Iceland, Israel: Asymptomatic	8.3	91.7	99.9	0.1	8.2	12.2
5	Spain, Israel	33.1	67.9	99.4	0.6	31.6	3.2
10		50.0	50.0	98.8	1.2	48.8	2.1
30	New York City	79.4	20.6	95.5	4.5	74.9	1.3
50	Almost Herd Immunity	90.0	10.0	90.0	10.0	80.0	1.3
70	Herd Immunity	95.5	4.5	79.4	20.6	74.9	1.3
90	Most Infected	98.8	1.2	50.0	50.0	48.8	2.1

* This is an illustrative table. There are no reliable estimates of disease prevalence in various countries.

## DISCUSSION

We showed that using COVID-19 testing to enable re-opening of airports may have its limitations and should be considered with caution. When diagnosing acute infection, it is important to avoid a false positive result that would unjustifiably quarantine a person and all persons that this person has met. It is equally important to avoid false negative diagnoses, because this can falsely reassure actual patients and hinder appropriate contact tracing and isolation. False positives and false negatives may have different implications for public health policy. Indeed, false positive test results may lead an actually healthy person to be wrongfully quarantined or retested, which is of cause inconvenient, infringing on personal freedom of travel, and somewhat costly. However, false negatives are much more damaging to society as a whole, as sick persons, by remaining undetected, can spread the disease in the community or on a plane. These effects should be instrumented into the assessment models, perhaps by adjusting the indices with appropriate (subjective) weights for the *PPV* and *NPV*.

The fraction of errors in diagnoses depends on the technical test characteristics, that is, the sensitivity and specificity, and the prevalence of the diseases in the population of interest. The prevalence can usually be obtained from national serological tests. Thus, a condition for planning a testing campaign for acute diseases is to use serological tests that estimate the prevalence of past infection.

For example, in the USA there are more than 400,000 people who have had COVID-19. This is approximately 0.1% of the population. Clearly, this is an underestimate of the true prevalence of the disease due to under-testing and under-reporting. If the prevalence of the disease in the USA is 0.1%, then even if the test is very accurate technically and has a specificity and sensitivity of approximately 90%, there is still a very low probability that a positive test is correct (*PPV*=0.9%), and approximately 99.1% of results are false positive. Even a prevalence of 1%, 10 times higher than the official estimate, would yield a *PPV* of 8.3% and 91.7% false positive results, with dire consequences to individuals. However, false negative results, and the risk of onboard infection, would be only 0.1%. Nevertheless, negative tests need to be interpreted with caution, taking into account the pre-test probability of disease (the prevalence) because false negatives may lead to spread of COVID-19.

The high number of tests that need to be performed to diagnose one person correctly, as shown in Table 4, may lead to high costs in any routine screening program.

The attempts to slow down the epidemic and control infections may lead to the use of tests with high sensitivity (and yielding fewer technical false negative results). However, we have shown that in a population with a low prevalence, these tests may lead to a high percentage of false positive diagnoses and a need for multiple tests at high cost to achieve a correct diagnosis.

We emphasize that in an RT-PCR test the RNA of the virus is detected, but not necessarily an infective virus. Thus, even correct test results may be false positive results, with respect to infectiousness.^20^ The prevalence of a disease is measured by serological testing. However, it can take up to five days for an infected person to develop antibodies to SARS-CoV-2, and thus the prevalence may be underestimated. Repeated testing can also improve the accuracy of the overall testing program.

We suggest caution in using COVID-19 testing in planning re-opening of airports. It seems prudent to recommend multiple testing of travelers with positive test results, together with obtaining information on previous exposure to persons with COVID-19 or being involved with populations that are known to have high prevalence of COVID-19. Using multiple quick and more accurate tests may be helpful before boarding an airplane.

## Abbreviations

COVID-19: coronavirus disease 2019
*fnf*: false negative fraction (in artificial population)
*FNF*: false negative fraction (in real population)
*fpf*: false positive fraction (in artificial population)
*FPF*: false positive fraction (in real population)
*J*: Youden index
*NND*: number needed to diagnose
*nns*: number needed to screen
*NNT*: number needed to treat
*NPV*: negative predictive value
*PPV*: positive predictive value
*PSI*: predictive summary index (Ψ)
*RD*: risk difference
RT-PCR: reverse transcriptase polymerase chain reaction
SARS-CoV-2: severe acute respiratory syndrome coronavirus 2.
